# Spatial omics technologies at multimodal and single cell/subcellular level

**DOI:** 10.1186/s13059-022-02824-6

**Published:** 2022-12-13

**Authors:** Jiwoon Park, Junbum Kim, Tyler Lewy, Charles M. Rice, Olivier Elemento, André F. Rendeiro, Christopher E. Mason

**Affiliations:** 1 Department of Physiology, Biophysics and Systems Biology, Weill Cornell Medicine, New York, NY USA; 2 Laboratory of Virology and Infectious Disease, The Rockefeller University, New York, NY 10065 USA; 3 The HRH Prince Alwaleed Bin Talal Bin Abdulaziz Alsaud Institute for Computational Biomedicine, Weill Cornell Medicine, New York, NY USA; 4 Caryl and Israel Englander Institute for Precision Medicine, Weill Cornell Medicine, New York, NY USA; 5 CeMM Research Center for Molecular Medicine of the Austrian Academy of Sciences, Vienna, Austria; 6 The Feil Family Brain and Mind Research Institute, Weill Cornell Medicine, New York, NY USA; 7 The WorldQuant Initiative for Quantitative Prediction, Weill Cornell Medicine, New York, NY USA

## Abstract

Spatial omics technologies enable a deeper understanding of cellular organizations and interactions within a tissue of interest. These assays can identify specific compartments or regions in a tissue with differential transcript or protein abundance, delineate their interactions, and complement other methods in defining cellular phenotypes. A variety of spatial methodologies are being developed and commercialized; however, these techniques differ in spatial resolution, multiplexing capability, scale/throughput, and coverage. Here, we review the current and prospective landscape of single cell to subcellular resolution spatial omics technologies and analysis tools to provide a comprehensive picture for both research and clinical applications.

**Supplementary Information:**

The online version contains supplementary material available at 10.1186/s13059-022-02824-6.

## Spatial omics technologies

**Fig. 1:**
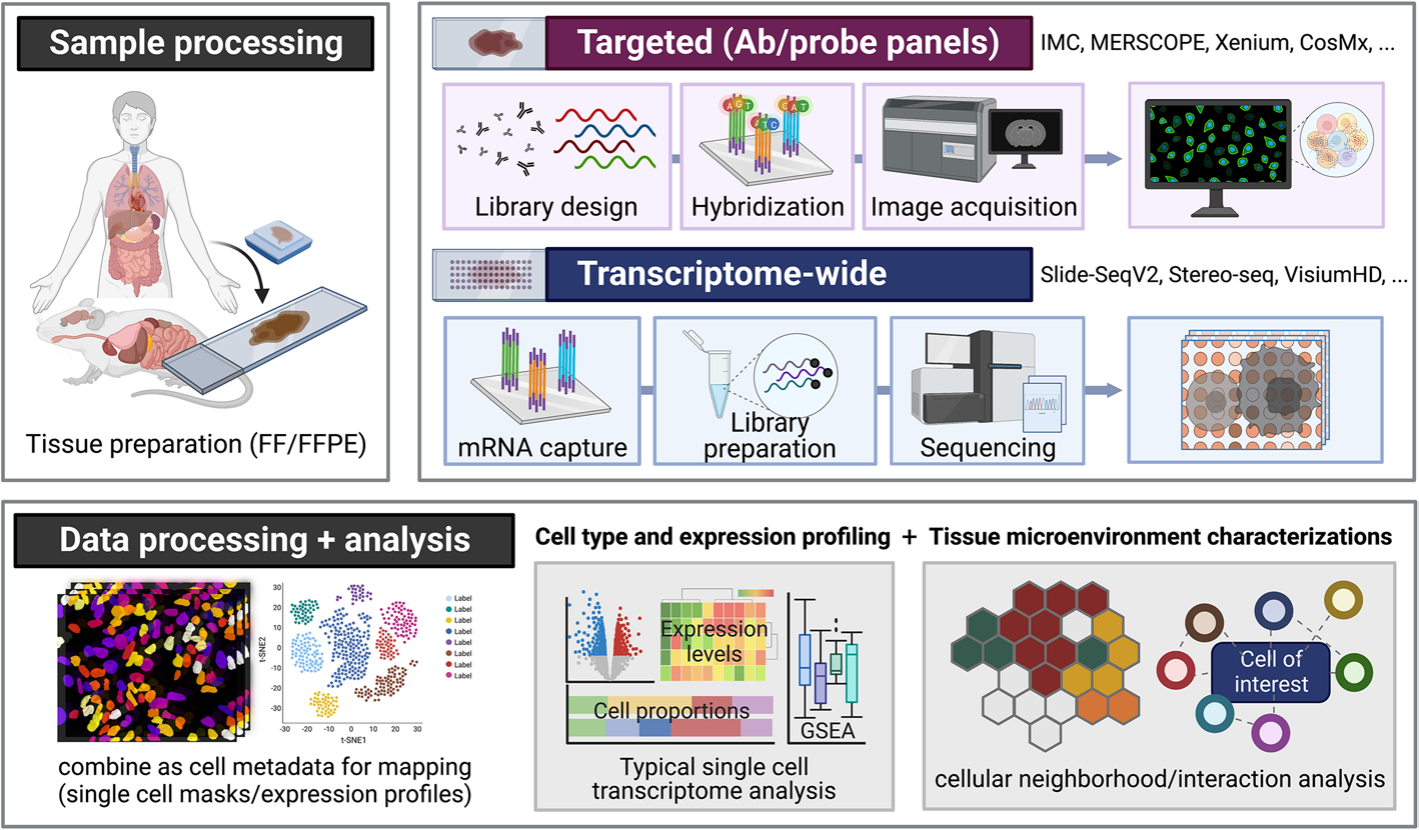
Typical workflow of spatial omics experiment. Most technologies offer compatibility with flash frozen or formalin-fixed paraffin embedded tissues. If in situ capture-imaging methods are chosen, a customized set of antibodies or probes will be hybridized to acquire or reconstruct images of multiple channels. With sequencing-based methods, barcoded regions within the slide will be captured for library preparation and sequencing. The downstream analyses are similar across technologies; once the signals are normalized, quantified, and pooled for each cell defined by the cell segmentation masks. Standard analyses such as differential expression, cell proportion, and gene set enrichment can be performed. Using spatial information in particular, cell–cell interaction and ligand-receptor analyses can be performed in-depth. Such information can also be used to define neighborhood or domains of tissue microenvironment, depending on the research question. Figure created with BioRender

Methods for molecular profiling of single cells in situ, within their native spatial context, are rapidly developing. In recent years, traditional experimental methods, including barcoding with reporters [1], immunohistochemistry (IHC) [2, 3], and fluorescent in situ hybridization (FISH) [4, 5], have given ways to spatial omics technologies to cover a larger number of transcripts or areas (Fig. 1). Broadly, spatial omics technologies vary in their spatial resolution (minimum size of molecular units profiled), coverage (breadth of tissue covered), scale and throughput (number of samples and profiling speed), and multiplexing capacity (breadth of molecular entities profiled simultaneously). Depending on the research question, the profiling methods can be divided into (1) targeted or multiplexed probe- or antibody-based and (2) transcriptome-wide or next-generation sequencing (NGS)-based approaches [6].

**Fig. 2:**
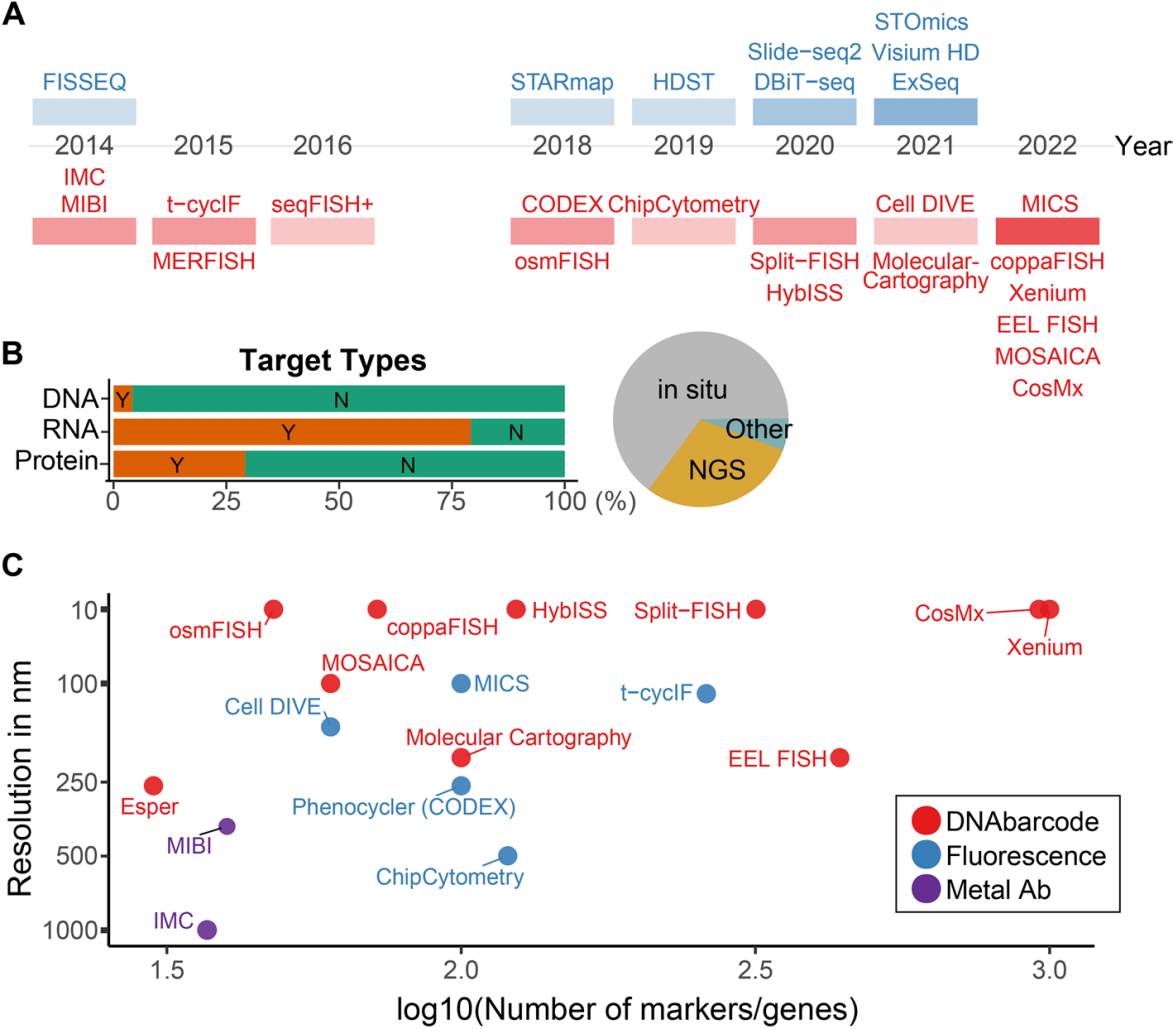
Timeline, type, and specifications of spatial omics technologies. **A** Timeline of all super resolution methodologies from 2010. Color intensity corresponds to the number of technologies published each year, and blue colored techniques are sequencing-based while red colored techniques are multiplexed IHC/IF methodologies. Top and bottom of the red boxes represent non-FISH and FISH based, respectively. **B** Proportions of target types and analysis approaches, where Y quantifies methods detecting given analyte (DNA, RNA, or protein). **C** Comparison of imaging-based technologies. Highest resolution that can be done, some of the super resolution microscopy-based methods were estimated at 50 nm and maximum number of the markers or genes that can be detected in an experiment. Note that depending on the technology, some of them were not optimized yet and can be expanded in future years. Excluded seqFISH + and MERFISH which claims up to 10,000 markers for detection (probe-based method)

Most spatial omics technologies with subcellular level resolution are performed on slide (in situ) using either microscopy or NGS platforms. Currently, there are over 50 different spatial mapping technologies available. In this review, we focus on the latest technologies that enable investigation of cells at the cellular and subcellular level (less than 10 μm, Fig. 2A). The history and technical workflow of lower resolution, non-single-cell, spatial omics technologies have been covered previously [7]. However, at < 10 μm resolution, the cell body and nucleus can be detected for single cell level quantification; with technologies that allow < 1 μm resolution, researchers can then detect a few other large organelles including cytoplasm-membrane distinction; at 200–300 nm, more well-resolved characterizations are possible including mitochondria-, ER-, or Golgi- specific transcript or protein quantifications. At 50 nm ranges, entirely new cellular phenotypes (e.g., movement of organelles and protein trafficking) can be measured. Table 1 highlights available technologies that allow subcellular capture of molecular entities in cells, as well as their technical specifications.

**Table 1:** Summary of single cell or subcellular spatial omics technologies. The table only includes single cell or subcellular resolution spatial omics technology, excluding spatial barcoding methods as it loses the organization context (e.g., CITE-seq, ZipSeq, ExSeq, XYZeq, and sci-space). MERSCOPE employs MERFISH as a platform solution. Slide-SeqV2 and DBiT-seq could be near single-cell. The plex numbers are based on the maximum validated or claimed in publications, as of March 2022. *Ab-based* Antibody-based methods (multiplexed spectrometry), *mFISH* Multiplexed FISH (probe-based methods), *NGS* Next-generation sequencing, *SR* Not specified and stated as subcellular resolution; falls within the capacity of resolution microscopy, *WT* Whole transcriptome

Technology	Type	Target	Resolution (nm)	Year	Company	Plex
FISSEQ	NGS	RNA	600	2014	FISSEQ	8742
MIBI	Ab-based	RNA, protein	350	2014	IonPath	40
IMC	Ab-based	RNA, protein	1000	2014	Fluidigm	37
ChipCytometry	Ab-based	Protein	500	2019	Canopy	120
MERFISH	mFISH	RNA	100	2015	Vizgen	10,000
CyCIF	Ab-based	Protein	110	2015	Rarecyte	261
seqFISH+^a^	mFISH	RNA	100	2016	Spatial Genomics	10,000
STARmap	NGS	RNA	SR	2018	N/A	1020
Phenocycler (CODEX)	Ab-based	RNA, protein	260	2018	Akoya	100
osmFISH	mFISH	RNA	SR	2018	N/A	48
Slide-seqV2^b^	NGS	RNA	10,000	2020	N/A	WT
HDST	NGS	RNA	2000	2019	N/A	WT
Split-FISH	mFISH	RNA	SR	2020	N/A	317
HybISS	mFISH	RNA	SR	2020	N/A	124
DBiT-seq	NGS	RNA, Protein	10,000	2020	AtlasXomics	WT
Stereo-Seq	NGS	RNA	500	2021	BGI STOmics	WT
ExSeq	NGS	RNA	1000	2021	N/A	WT
Molecular Cartography	mFISH	RNA	200	2021	Resolve Biosciences	100
Cell DIVE	Ab-based	Protein	SR	2021	Cytiva	60
CosMx^c^	mFISH	RNA, protein	50	2022	NanoString Technologies	960
Xenium^c^	mFISH	RNA, protein	50	2022	10X Genomics	1000
Visium HD	NGS	RNA	5000	2021	10X Genomics	WT
Esper	mFISH	RNA	260	2021	Rebus Biosystems	30
Seq-scope	NGS	RNA	600	2021	N/A	WT
PIXEL-seq^c^	NGS	RNA	1000	2021	N/A	WT
MOSAICA	mFISH	RNA	100	2022	N/A	60
MICS	Ab-based	Protein	100	2022	Miltenyi Biotec	100
EEL FISH^c^	mFISH	RNA	200	2022	Rebus Biosystems	440
coppaFISH^c^	mFISH	RNA	SR	2022	N/A	72

^a^updated version of seqFISH; ^b^updated version of Slide-seq

^c^In preprint or not publicly available yet

### Targeted spatial omics methods using antibodies and RNA probes

Targeted spatial omics methods are appropriate when there are specific molecular entities of interest to identify cellular states, identity, and function. Antibodies and RNA probes, such as endogenous transcripts or proteins, are the most ubiquitous detection methods (Fig. 1). Traditional immunofluorescence (IF) imaging methods can only capture four to five channels at a time, limited by spectral overlap. Using probes or antibodies that contain cleavable linkers for sequential imaging and/or barcoding scheme to distinguish multiple probes imaged simultaneously or within the same wavelength, now researchers can profile more than 50 cellular entities from a single slide. The technologies using antibody panels with sequential rounds of staining, imaging, and bleaching/stripping include: Cyclic ImmunoFluorescence (CyCIF) [8], iterative bleaching extends multiplexity (IBEX) [9], multi-epitope-ligand-cartography (MELC) [10], iterative indirect immunofluorescence imaging (4i) [11], ChipCytometry [12], Cell DIVE (multiplexed immunofluorescence, MxIF) [8], and MACSima Imaging Cyclic Staining (MICS) [13]. These are generally limited to 30–50 targets due to constraints in tissue integrity, spectral overlap, and spillover (Table 1). To further increase the number of antibodies, technologies such as CO-Detection by indiEXing (CODEX) [14] increase the target breath to 100 by staining a cocktail of DNA-barcoded antibodies. It then uses complementary oligonucleotide probes and comparatively gentle iterative hybridization cycles for detection. Alternative to imaging-based readouts are methods which use mass spectrometry, such as imaging mass cytometry (IMC) [15] and multiplexed ion beam imaging (MIBI) [16]. For IMC and MIBI, antibodies are tagged with rare metals, and tissue samples are stained with the whole antibody cocktail. The tissue sample is then ablated with an ionizing laser in a raster fashion, and detection of the rare metals is possible through time-of-flight mass spectrometry, which has much less spectral overlap than optical methods.

To avoid nonspecific binding, epitope loss, and tissue degradation, antibody-methods may require further optimization, such as permeabilization or antibody incubation protocols. Using pre-optimized, commercially available antibody panels can significantly reduce this process, although this option is not available for all tissue types. Virtually, all antibody methods capture regions of interest (ROIs) within the tissue slide due to constraints of acquisition time (optical methods) or cost (mass spectrometry-based methods), while providing data that is only relatively quantitative (quantified by relative spectral intensity). A detailed description of these technologies is covered in related manuscripts and reviews specifically focusing on multiplexed methods [17–19].

Similar to antibodies, mRNA probes (specific for sequences of targeted transcripts) can be used to design a panel for targeted spatial profiling. Such approaches have shorter protocols, easier handling, and enable more transcripts (generally few hundreds of transcripts, up to 10,000) to be captured than antibody-based methods [20]. Different multiplexed RNA FISH methods use slightly different approaches to probe design, imaging, and stripping [21]. Specifically, high multiplexing is achieved by using spectral barcoding (specific combination of fluorophores each targeting segments of an RNA resolved by microscopy) [22] and temporal barcoding (multiple rounds of probe hybridization and stripping to create predefined color sequence) [23]. Recently, the combination of two barcoding methods and more complicated barcoding strategies can increase the number of molecular entities that can be profiled. For example, while seqFISH uses spectral barcoding of genes across four or five fluorophores for each given temporal barcode, seqFISH + adds mRNA-specific sequences to assign a pseudocolor for each spectral barcode (fluorophore). The new seqFISH + technology can now capture 60 pseudocolors instead of 5 per cycle, allowing more than 8000 gene profiles [24].

Barcoding approaches can generally capture more transcripts than antibody-based methods, but are more sensitive to losses (false-negative signals due to dropout) and tissue degradation between the cycles (Fig. 2B, C) [21]. To overcome potential errors and dropouts, most probe-based methods utilize extra hybridization steps added for every few cycles to ensure the rest of the transcripts are detected properly. Current technologies include multiplexed error robust fluorescence in situ hybridization (MERFISH) [25], molecular cartography [26], sequential fluorescence in situ hybridization (seqFISH +) [24], sequent ouroboros single-molecule FISH (osmFISH) [27], Split-FISH [28], hybridization-based in situ sequencing (HybISS) [29], Esper [30, 31], CosMx [32], Multi Omic Single-scan Assay with Integrated Combinatorial Analysis (MOSAICA) [33], and combinatorial padlock-probe-amplified FISH (coppaFISH) [34]. Each technology utilizes slightly different probe length, design methods, and protocols. For example, MERFISH probes hybridize with disulfide bond while seqFISH uses DNAse I treatment to cleave the probes for the following rounds of detection. Although most technologies would give similar results in most cases, factors such as probe length, tissue autofluorescence, tissue degradation, and protocol compatibility need to be considered for optimal results. For example, tissues can have different stability for signals over multiple rounds (or long duration) of enzymatic treatments or temperature variations. Indeed, prior work has shown a range of concordance between various spatial imaging technologies, such as with the GeoMx and Hyperion systems, which showed high correlation for differences in cell types and expression of healthy vs. pneumonia (*r* = 0.699) and different stages of COVID-19 (*r* = 0.630) but lower correlation of expression metrics of COVID-19 vs. healthy patient (*r* = 0.362) [35].

In general, the probe-based methods are more quantitative than antibody-based methods, as absolute numbers of transcripts are counted as individual dots within each scanned spot. Some probe-based methods can also utilize oligo-conjugated antibodies to incorporate subcellular protein landmarks (i.e., organelles) as well. However, the probe design and library composition are critical and sometimes limiting; some techniques are limited by gene length (transcripts must be > 750 bp), expression levels (highly expressing transcripts can create optical crowding and zero inflation spots called), and isoforms (splicing frequencies may be a source of error but can also target differentially transcribed exons or introns) [36]. Some of these limitations are an active field of research and can be overcome in several ways if properly accounted for, such as decreasing the binding range (e.g., 25 nucleotides) or overlapping probes to map isoforms. Despite these limitations, there are more published data and analytical software tools already optimized for these targeted technologies (vs. transcriptome-wide methods, described below).

### Transcriptome-wide spatial omics with NGS platforms

In addition to scRNA-seq methods that allow a comprehensive view on the transcriptome of a cell, several methods for spatial profiling of the transcriptome in an unbiased manner have been developed. Earlier methods focused on targeted acquisition of sample subsets for sequencing and used laser-capture microscopy (LCM) and photocleavable marker-based methods, where specific regions marked by surface markers or mRNA probes were collected and sequenced. LCM-seq [37], geographical position sequencing (Geo-seq) [38], NICHE-seq [39], and NanoString GeoMx DSP [40] are a few additional contemporary examples of this technology. These technologies allow user-directed profiling of specific ROIs with as few as 10 cells, allowing researchers to characterize multiple replicates or tissue types/locations for each sample.

Methods that allow single cell or subcellular resolution rely on spatial barcoding and in situ sequencing. Technologies such as fluorescent in situ sequencing (FISSEQ) [41], spatially resolved transcript amplicon readout mapping (STARmap) [42], Slide-SeqV2 [43], deterministic barcoding in tissue for spatial omics sequencing (DBiT-seq) [44], expansion sequencing (ExSeq) [45], high-definition spatial transcriptomics (HDST) [46], Seq-scope [47], polony (or DNA cluster)-indexed library-sequencing (PIXEL-seq) [48], and SpaTial Enhanced REsolution Omics-Sequencing (Stereo-Seq) [49] (and ST, 10X Visium for multi-cell version of the same technology) use grid-like nanoballs or sequencing sites on the slide. The resolutions and chemistry of the sequencing sites vary by technologies, as well as amplification methods. For example, FISSEQ uses rolling cycle amplification (RCA) where a random hexamer reverse transcription (RT) primer gets hybridized for cDNA transcription and amplification; STARmap uses padlock probes to avoid RT and use of RNA template; technologies like HDST, Slide-SeqV2, and DBiT-seq all use Barcode and UMI structures with varying nucleotide lengths optimized for each technique and preferred sequencing platform. The individual spots on these slides are several times smaller than typical mammalian cells, enabling single to subcellular characterization of sequencing reads. Although it may be more costly, in situ sequencing approaches allow whole-slide detection whereas capture-based sequencing methods are more focused on smaller ROIs within the tissue. Because the resolution can be limited by the size of the amplicon hybridized on the slide for sequencing, different strategies to improve the UMI and quality of the reads are currently being explored in these technologies. Also, methodologies to capture total transcriptome (viral, coding, and noncoding RNAs) are also developed, such as spatial total RNA-sequencing (STRS) [50].

In addition, there are spatial barcoding methods such as CITE-seq [51], ZipSeq [52], ExSeq [45], XYZeq [53], or sci-space [54], where additional cellular information such as antibody staining or barcoding is added before pooling for single cell sequencing workflow. Additional single cell features such as cell types or tissue location or compartmentalization can be deduced; however, a precise picture of cellular organization is not yet possible. Inspired by these technologies and to overcome the limitations, multiplexed detection methods of transcriptome and proteome have been developed. Examples include spatial multi-omics (SM-Omics) [55], Spatial PrOtein and Transcriptome Sequencing (SPOTS) [56], and spatial co-indexing of transcriptomes and epitopes for multi-omics mapping by NGS (spatial-CITE-seq) [57]. These technologies combine existing NGS-based methodologies to allow computational reconstruction of spatial full transcriptome and 200 + proteome maps. Although these technologies make use of NGS technologies to cover the full transcriptome while recording large panels of proteins in tissues, the full transcriptome characterizations are still limited by resolution (SM-Omics uses 10X Visium which allows 55 μm resolution), or location detection (SPOTS and spatial-CITE-seq uses CITE-seq, which is an antibody-binding based method, to provide cellular context, not precise locations within the tissue).

### Other spatial omics technologies for multi-modal study

Similar to the spatial omics technologies introduced above, which mainly focus on gene expression profiles (and surface marker proteins), approaches around spatial genomics, metabolomics, metagenomics, and epigenomics are also emerging. Spatial ATAC sequencing can be performed by in situ Tn5 transposition, and probe ligation using microfluidics devices, followed by standard digestion and sequencing for chromatin accessibility profiling [58, 59]. Resolution is limited by the microfluidic channel width (20 μm); however, single cell resolution is less crucial than transcript quantification as the analysis relies on the signals within the nuclear regions. Similarly, specific chromatin modifications can be quantified using Spatial-CUT&Tag that applies CUT&Tag chemistry with microfluidic devices [60]. Both technologies use deterministic barcoding delivered over the tissue surface through a microfluidic device attached to the slide. The barcodes are delivered twice perpendicularly so that the combinations result in 2D arrayed pixels containing spatial information. Spatial metabolomics techniques such as targeted approaches using antibodies (metaFISH) or untargeted using matrix-assisted laser desorption/ionization imaging mass spectrometry (MALDI-IMS) hold promise for mapping the spatial context of metabolic species and molecular interactions within the native tissue context, but still suffer from trade-offs in spatial resolution or the breadth of molecular entities profiled [61].

Such added layers of genomic data allow researchers to ask new biological questions. For example, in addition to expression level changes within tissue microenvironment, clonal expansion of specific mutations and spatial co-occurrences can be investigated using spatial genomics such as slide-DNA-seq [62]. This method is a modified version of slide-seq where DNA sequences are captured with small (3 mm) beads that are spatially indexed, instead of RNA transcripts. Optimized methodologies for histone removal and Tn5 treatments for a variety of tumor tissue types have been shown to prevent potential bias in DNA capture. More recently, spatial host-microbiome sequencing (SHM-seq) has been reported [63]. SHM-seq is an adapted version from Spatial Transcriptomics, where mRNA probes for transcript captures are modified to DNA capture probes so that they can obtain both polyadenylated transcripts and 16S rRNA hypervariable regions. The recent progress on spatial characterizations allows researchers to locate interactions at the genomics, cellular, and organismal level [64].

## Overall workflow and experimental design criteria

### Sample preparation and experimental design

**Fig. 3:**
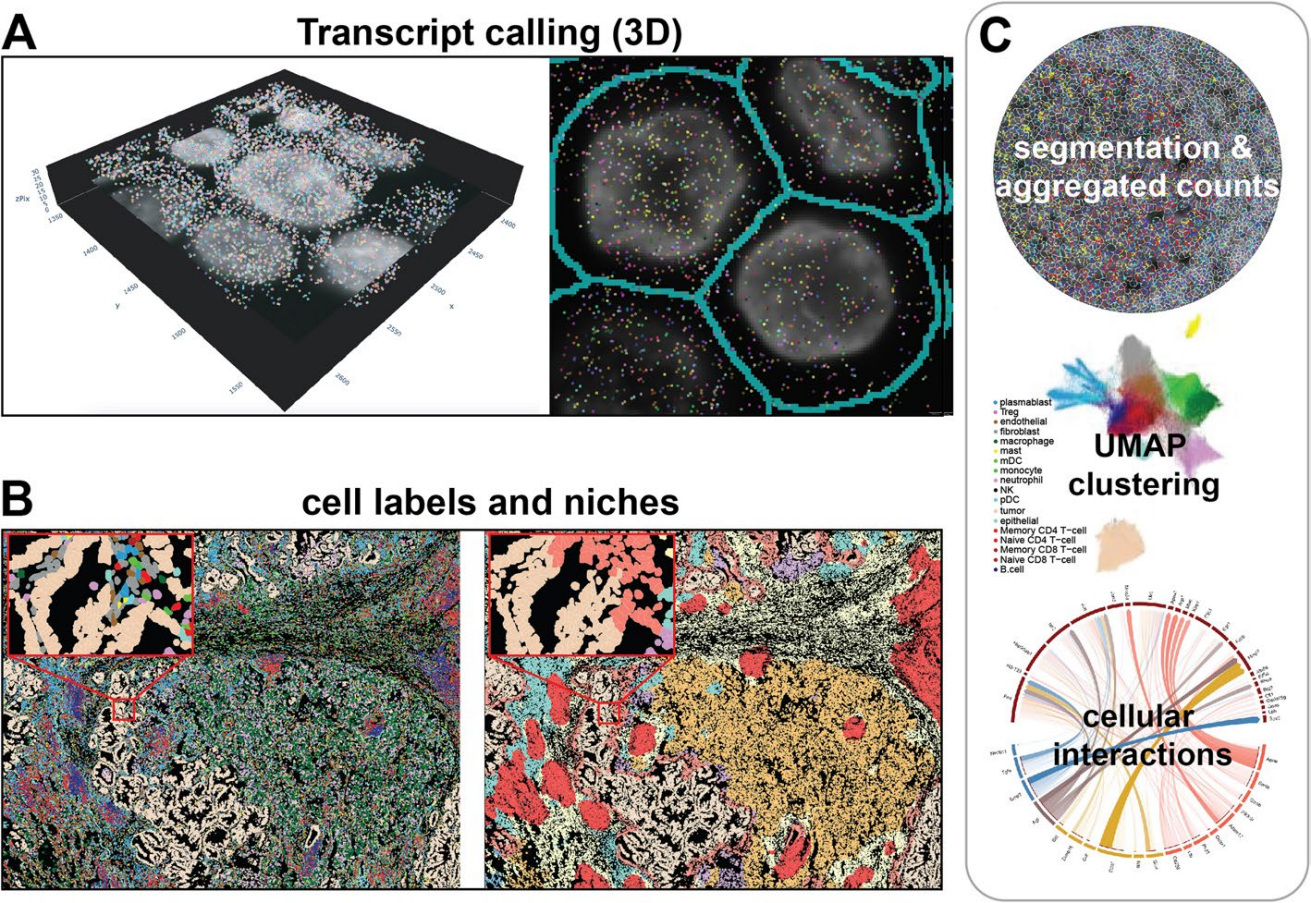
Example subcellular spatial omics data and analysis. **A** Subcellular spatial omics analysis starts with calling transcripts (or other molecular entities of interest) at their 3D locations from z-stacked images. **B** Using boundaries drawn by cell segmentation algorithms, the counts are aggregated into single-cell expression profiles, from which cell labels and neighborhood traits are inferred. **C** Both information can then be used for analyses commonly used for single cell and bulk image and sequencing methods, such as differential expression, clustering, cellular interaction analyses. The tissue sample images in all panels were provided by NanoString Technologies (CosMx platform) [65]

Overall experimental and analytical workflows span a few consistent features (Fig. 1). Generally, mouse or human tissue slides are used for experiments, since the relevant probe and target libraries are already commercially available and represent the largest market; however, in principle these methods can work on any species with an annotated genome (Fig. 3). It is also possible to use cell cultures or sections from specific culture platforms. Most of the techniques offer compatibility with flash frozen (FF) and formalin-fixed paraffin embedded (FFPE) formats. FF format often yields better RNA quality and simpler extraction processing; however, FFPE format more faithfully conserves tissue architecture and is easier to store and ship. Success also varies depending on the sample quality (RNA integrity and processing protocols) and technology (probe/antibody design, permeabilization and chemistry for hybridization, imaging, and library preparation). Some technologies generate stacked multiplexed images to capture all the transcripts across 5–10 μm thick sections and to differentiate transcripts expressed in nuclear and cytoplasmic regions [26].

A key question to consider in choosing the methodology and the experimental design is whether specific transcripts, pathways, or cell types need prioritizing. If specific loci are already known, then choosing an in situ capture based methodology and obtaining absolute quantification of the transcripts is often the better approach. Alternatively, if the research hypothesis is more discovery-based, especially for unknown cellular subpopulations, or if a project is aiming for a global comparison across conditions or samples, NGS-based methods may be more appropriate.

### Available reference databases for integration and validation

Even though the field is relatively new, there are some reference data sources available to explore different methodologies and to use as healthy references (Table 2). For example, the GeoMx spatial organ atlas offers human tissue spatial data from five organ systems: kidney, brain, intestine, lymph node, and pancreas (https://nanostring.com/products/geomx-digital-spatial-profiler/spatial-organ-atlas/). Vizgen also released a mouse liver spatial atlas for people to explore and understand the extent of the technologies, and Akoya biosciences has shared its data from a range of tissues online, including brain, placenta, breast, lung, head and neck, and tonsil online (https://www.akoyabio.com/fusion/data-gallery/). Since many of these investigations focus on cancer samples and normal controls, some datasets can be accessed through human tumor atlas (https://data.humantumoratlas.org/) or the Human Biomolecular Atlas Program (HuBMAP, https://portal.hubmapconsortium.org/).

**Table 2:** List of reference atlas available for spatial omics applications (as of March 2022). *HPA* Human Protein Atlas (https://www.proteinatlas.org/), *HCA* Human Cell Atlas (https://www.humancellatlas.org/), Tabula Sapiens (https://tabula-sapiens-portal.ds.czbiohub.org/)

	**GeoMx**	**HPA**	**HCA**	**HCA**	***Tabula sapiens***
		Coverage	Project counts	Est. cell counts	Cell counts
Lung	N	Y	18	6,200,000	35,682
Kidney	Y	Y	9	4,900,000	9641
Liver	N	Y	10	5,400,000	5007
Brain	Y	Y	14	9,700,000	N/A
Skin	N	Y	12	1,500,000	9424
Colon (large intestine)	Y	Y	4	1,100,000	N/A
Rectum (large intestine)	N	Y	2	372,300	N/A
Small intestine	N	Y	3	986,700	12,467
Stomach	N	Y	3	4,600,000	N/A
Lymph node	Y	Y	3	536,700	53,275
Prostate	N	Y	3	798,900	16,375
Ovary	N	Y	3	525,100	N/A
Uterus	N	Y	3	1,000,000	7124
Heart	N	Y	8	6,200,000	11,505
Esophagus	N	Y	4	914,600	N/A
Bone marrow	N	Y	3	571,700	12,297
Bone (skeletal muscle)	N	Y	2	212,600	N/A
Adipose (fat)	N	Y	4	1,000,000	20,263
Bladder	N	Y	2	486,700	21,517
Eye	N	Y	7	4,900,000	10,650
Testis	N	Y	2	30,200	N/A
Spleen	N	N	5	4,800,000	34,004
Appendix (large intestine)	N	Y	N/A	N/A	N/A
Bile duct	N	N	1	84,375	N/A
Pancreas	Y	Y	16	5,100,000	13,497
Smooth muscle	N	Y	N/A	N/A	30,746
Nasal cavity	N	N	3	114,800	N/A
Peritoneum	N	N	N/A	N/A	N/A
Blood	N	N	28	3,000,000	50,115
Breast	N	Y	2	709,100	N/A
Large intestine	N	Y	2	482,300	13,680
Mammary gland	N	N	1	N/A	11,375
Salivary gland	N	Y	1	N/A	27,199
Thymus	N	Y	3	4,800,000	33,664
Tongue	N	N	1	N/A	15,020
Trachea	N	N	3	564,700	9522
Vasculature	N	N	1	N/A	17,071

Depending on the publications and technologies, some researchers have made data explorer websites or share semi-processed count data on public repositories [66–68]. Currently, systematization of data standards is lacking, except for the convergence of imaging data into the metadata-rich OME-TIFF file format. Public sharing of datasets and in-depth, online guides will further aid the community by ensuring standardization and reproducibility of data [69].

Alternatively, single cell and single nuclei RNA sequencing can be used to cross validate and overcome limitations of spatial omics technologies (i.e., limited detection of rare transcripts, interpolation of missing transcripts through cell type label transfer). Commonly used reference databases are summarized in Table 2. When choosing the reference, it is crucial to understand tissue type and technology of interest. Sample types, tissue dissociation methods, cellular heterogeneity, and type of sequencing chemistry can carry considerable impact, particularly when interested in rare cell types or transient cell expression states (e.g., fetal hemoglobin). For more rigorous data integration and validation, common coordinate framework (CCF) based mapping has been discussed and developed [70, 71].

## Computational methodologies to analyze spatial omics data

The information captured from spatial omics data at subcellular resolution is predominantly converted into single cell format (quantification of counts/cell) or csv files, which are then suitable for downstream analysis. In the following section, we introduce methodologies and published software packages for general spatial analysis and available multiplexed image viewers for visualization of the highly multiplexed images. We also review key methods involving traditional analysis similar to scRNA-seq analyses, newer methods that make use of spatial information, and auxiliary methods applied directly to the raw image level.

### Available general-purpose software and pipelines

As most recent spatial omics technologies support more markers than traditional image formats support, visualizing results of spatial omics data is a non-trivial task. Several specialized image software such as ImageJ or napari can be used to visualize subsets of markers. As some spatial platforms gather information on a per spot basis, several denoising software tools such as content-aware image restoration (CARE) [72], residual channel attention networks (RCAN) [73], and Noise2Void [74] can be applied to raw images to improve the quality especially in fluorescence based platforms. For mass cytometry based spatial platforms, data quality can be improved by spillover correction software [75]. For example, if the ground truth of spillover is known or empirically adjusted from the data, Reinforcement Dynamic Spillover EliminAtion (REDSEA) [76] leverages the spatial proximity of cells with signal that comes from generally mutually exclusive markers.

There are also user-friendly end-to-end software packages for spatial omics. Graphical user interface supporting software pipelines like ImageJ [77], QuPath [78], CellProfiler [79] and many other command line tools take raw input data and transform them into single cell format with coordinate information. Other software like squidpy [80] and scimap (https://github.com/labsyspharm/scimap) provide user-friendly Python APIs for spatial analysis, visualization, and a collection of preprocessed datasets from multiple diverse spatial platforms in AnnData format. Depending on the platform, some technologies offer end-to-end packages for sample processing to data visualization and analysis. For example, TITAN [81] offers open-source software options for IMC image visualization, cell segmentation, analysis, and export. Other commercial technologies also offer end-to-end solutions optimized for each technology, such as AtoMx for GeoMx and CosMx (NanoString), PhenoCycler Software Analysis Suite for CODEX and PhenoCycler (Akoya), and MERSCOPE Visualizer for MERFISH platform (Vizgen).

### Traditional spatial omics methods for single cell analysis

Typically, the outputs of most spatial omics analyses involve images or barcodes with spatial coordinate information. Such spatial information can be treated as an additional layer of metadata and help inform downstream analysis. Successful downstream analyses on spatial omics data, which either are high dimensional images or spatial barcodes with coordinates, heavily rely upon accurate cell segmentation, which is a process to infer cell boundaries based on intensity values from captured coordinates. Cell segmentation is done on generalizable cellular features, such as DNA staining for nuclei and membrane staining or (cell-type specific) cytoplasmic markers for cell boundaries. Some of imaging-based methods use serialized, z-stack images to better distinguish cellular features each round of imaging; however, this is not so common in sequencing-based methodologies because imaging for segmentation is typically done right before the library preparation or on consecutive tissue slices.

There are two broad categories of cell segmentation algorithms both based on supervised learning: (1) a combination of computer vision-based feature extraction and machine learning models like random forests and (2) convolutional neural network (CNN)-based deep learning models. The first group, including Ilastik [82], extracts intensity, edge, and texture features from images using Gaussian filters and their derivatives. Then, the user labels pixels in images typically representing nuclei, cytoplasm, and background areas, which are used as labels for pixel classification using random forests based on the extracted features. The software can then either produce probability maps for the specified classes which can be segmented with CellProfiler [79] or a segmentation map directly.

The other group uses deep learning for segmentation. DeepCell [83], Stardist [84], Splinedist [85], Cellpose [86], and Omnipose [87] are all convolutional neural network (CNN)-based models capable of feature extraction, probability map prediction, and cell segmentation. Deepcell incorporates multiple segmentation subtasks including cell segmentation, nuclei segmentation, and cell tracking for sequence of moving cell images via deep watershed methods. DeepCell also offers Panoptic net-based models that use a combination of semantic and instance segmentation. Related tools like Stardist and Splinedist focus on nuclear segmentation using the geometric properties of nuclei; Stardist models nuclear morphology as star convex polygons and assigns probabilities based on center-to-border distances to detect and separate objects, and splinedist generalizes a similar model to capture more smooth-shaped cell masks and recognize eccentric cells using spline interpolation. Cellpose is a general-purpose model for both nuclear and whole-cell segmentation, while the more recent Omnipose specializes in segmentation of bacterial cells that often are elongated or eccentric. Regardless of the segmentation method, the various analytical approaches converge on the creation of a single-cell matrix analogous to scRNA-seq data, by aggregating RNA/protein signal intensity values by sum or mean based on the segmentation masks.

The aggregated single-cell matrix can then be transformed and normalized through a combination of scaling functions such as logarithm (base2 or base10), minmax, or *z*-score, and they can be batch corrected using algorithms such as Combat [88], MNN Correct [89], batch-balanced k-nearest neighbors [90], and harmony [91]. One can apply conventional scRNA-seq analysis such as dimensionality reduction using PCA [92], T-SNE [93], or UMAP [94] and clustering with Leiden [95] or Phenotyping by Accelerated Refined Community-partitioning (PARC) [96] algorithms. The neighborhood of the cells can also be defined from the intensity features, using modern graph based cell clustering methods such as Louvain [97], Leiden, or PARC clustering.

From the expression profiles, phenotyping is possible by manually assigning cell labels based on the expression of markers. While this procedure may be laborious, it enables the detection of novel cell types and states. Nonetheless, methods for automated prediction of cell type identities such as Astir and Stellar also exist [98, 99]. Single-cells and their phenotypes can be visualized either by projecting feature intensity as colors on scatter plots of reduced dimensionality projections, or by visualizing features on heatmaps at the single-cell or cell type level. Common downstream analysis often involves the comparison of cell type abundance between samples of contrasting biological groups and detection of differential expression of markers within cell types between sample groups. Several of these methods were reviewed previously [100].

For quantitative technologies, expression levels can be also compared using standard differential gene expression analysis for bulk and single cell RNA-seq. In addition to differentially expressed genes, spatially variable genes can be obtained by published packages such as Trendsceek [101], SpatialDE [102], Spatial PAttern Recognition via Kernels (SPARK) [103], and SpaGCN [104]. These packages use different algorithms to compute spatial variations and are implemented for specific technologies. For example, SpatialDE uses Gaussian process regression in which it can detect genes across regions or multiple conditions but is computationally intensive and cannot identify domains. SpaGCN uses a graph convolutional network (GCN) and provides both gene and domain level spatial analyses, but tissue structures identified by cell types are not included in the analysis unlike the methodologies covered in the next section. Also of note, the methods for normalization, batch correction, and data cleaning are just as paramount in spatial expression mapping as they are in bulk analysis, and a range of methods (e.g., COMBAT, EDAseq, RUV2, SEER, etc.) can be used [105].

### Novel spatial omics methods using spatial information

Highly resolved spatial omics data allows discovery of new cell types, cell interactions, and tissue structures compared to traditional, single cell sequencing methods because the data comes with paired spatial information in the native tissue conformation. Currently, most methods in this area construct cell graphs, with cells as nodes and edges based on threshold spatial distance between cells to leverage spatial information. The two large branches in this field are (1) spatial microenvironment analysis, which groups and analyzes cells based on their spatial context and (2) inference of intercellular interactions, which investigates how frequently a pair of cell types interact in native tissue conformation.

One major lineage of computational spatial omics uses cell context to perform various downstream tasks. Stellar (https://github.com/snap-stanford/stellar) is a cell type annotation tool using both marker expression profiles and spatial context information. By learning cellular phenotypes not only from the intensity of markers but also from the spatial arrangement of cell types, Stellar enables the prediction of cell types in an unlabeled dataset and discovery of cell types specific to a new tissue. SpatialLDA [106] is a tumor microenvironment detection method that identifies associated topic or context of each cell based on cell type distribution of immediate spatial neighbors, which for tumor cells could be thought of as tumor microenvironments. UTAG [107] is a structural microanatomy annotation and analysis method that categorizes cells into anatomical structures across organs and diseases including cancers. Both SpatialLDA and UTAG infer larger-scale patterns or organization in tissue, which can be further interrogated to understand how cellular composition and interaction give rise to tissue structure capable of contributing to organ-specific physiology, overall organ architecture, or micro-environments in the tumor micro-environment which may condition clinically relevant outcomes.

Another branch of interest specifically focuses on learning intercellular interaction. Boisset et al. introduced a computational foundation to quantify cell-to-cell communication by graph permutation test [108]. They proposed assessment of intercellular communication frequency by randomly mixing cell type identities and assigning a statistical likelihood to empirically observed interaction between cell types. A similar approach, using permutation analysis, has been used to detect increased interaction between macrophages and fibroblasts in alveolar walls, which potentially explains fibrosis and the thickening of the alveolar wall in COVID-19 patients [35, 67, 68]. The increased macrophage interactions with fibroblast and other immune cells were consistently observed when cellular interaction clusters were defined by co-occurrence of the cell type proportion changes (https://github.com/jpark-lab/SpatialAnalysis). Other methods try to explain cellular communication through machine learning methods. For example, node-centric expression modeling (NCEM) [109] models cell-to-cell communication events by gradient analysis of variational graph autoencoders. The idea here is to investigate which change in cell-to-cell communication results in a change in observed gene expression through a non-linear graph neural network. Multiview Intercellular SpaTial modeling framework (MISTy) [110] is a graph-based method that investigates marker (gene) networks at multiple spatial resolutions to investigate intra- and inter- cellular interaction at multiple lengths.

With the ability to capture molecules at a subcellular resolution, co-localization and compartmentalization of molecular entities can be analyzed at a deeper level. For example, RNA species in different subcellular compartments (i.e. endoplasmic reticulum and nuclear vs. cytoplasmic) and their spatial patterning within the cell can be extracted as an independent feature from expression level [20]. Transcripts that are dependent on cellular states such as infection, cell cycles, circadian rhythm can be profiled more accurately and provide new insights. In addition, distribution of cellular features (i.e., protein or viral RNA transcript patterns within a cell) can be studied for biological significance. These features are limited by our understanding of cellular processes and by methodologies to analyze such changes and generate hypotheses in an unbiased manner.

### Multi-modal analysis and ML-aided spatial data analysis methods

Integration of spatial omics data with other data modalities, such as single-cell RNA or assay for transposase-accessible chromatin (ATAC) sequencing, can enable an even more comprehensive view of cellular systems, by complementing the spatial assays in terms of the number of molecular entities under study and cells profiled. A popular approach to integrate different datasets consists of identifying a subset of variable or “notable” features to serve as anchors across two data modalities. Several methodologies were developed around the integration between different single cell or single nuclei sequencing modalities such as RNA and ATAC. Multi-Omics Factor Analysis (MOFA) introduces a statistical framework for the integration of data modalities, specifically within a common sample space derived from the same sets of cells [111].

One of the most used software packages for scRNA-seq analysis, Seurat, also has developed methodologies to integrate such modalities as well as antibody-derived tags from cellular indexing of transcriptomes and epitopes by sequencing (CITE-seq) and spatial omics technologies such as Visium, by using weighted nearest neighbors (WNN) analysis. Multimodal omics analysis framework (MUON), which introduces the MuData format compatible across Python, R, and Julia programming languages, provides a shared interface for commonly used methodologies such as MOFA, WNN, and similarity network fusion (SNF) [112]. Similar framework packages include MultiMAP [113], linked inference of genomic experimental relationships (LIGER) [114], inteGrative anaLysis of mUlti-omics at single-cEll Resolution (GLUER) [115], clustering on network of samples (Conos) [116], and integrative non-negative matrix factorization (iNMF) [117], but some of the packages are more focused on specific spatial omics technologies or analysis of single-cell sequencing modalities. Recently, more packages and methodologies, such as Cell2location [118], CellTrek [119], multi-modal structured embedding (MUSE) [120], and Tangram [121], are being developed specifically to map single cell information to spatial omics analyses. For example, Tangram aligns expression profiles from sc/snRNA-seq to spatial datasets from the same region including MERFISH, STARmap, general smFISH, Visium, and histological images [121]. Such methods to map other data modalities are also used within spatial omics dataset when gene imputation, an approach used to fill in the missing datapoints due to low detection level, limited number of targets, potential errors or dropouts, or genetic variation are needed [122]. On the contrary, multi-omics image integration and tissue state mapping (MIAAIM) focuses on integration of different spatial omics modalities that have diverse densities and spatial resolutions [123]. As most of these software packages only offer methodologies for proper integration of multiple data modalities without losing single cell resolution, more work is needed for multimodal data exploration and visualization.

## Conclusion

As spatial omics technologies mature and provide a deeper understanding of the cellular states and functions, spatial epigenomics, metagenomics, and metabolomics will also reveal a great number of biological insights that complement the transcript-level findings. Integration strategies across different molecular classes (for example, integrating metabolomics data with proteomic, transcriptomic, or genomic data) would also be needed. Development of appropriate analysis packages that offers end-to-end solutions for each technology, as well as compatibility with orthogonal platforms is also crucial to increase the usage and application of these technologies.

In summary, the spatial omics field has blossomed and radically increased the breadth and resolution of in situ experiments in the past few years. These technologies can now boast detection of more than 10,000 unique gene targets with 50–100 nm spatial resolution. Developments in probe chemistry, image acquisition, and commercialization are driving down costs, transforming spatial omics technologies into a commonplace technique available to all labs, similar to NGS in the 2010s and microarrays in the early 2000s. This unparalleled depth and richness of data leading to spatially intact single cell profiles promises to fuel new discoveries for infectious disease, tumor oncology, and basic science applications like cell signaling, migration, and spatiotemporal-delineated functions. Efforts to introduce three-dimensional, deep-slide scanning, and time series data collection will also continue to propel the field even further in the coming years, revealing novel cellular architectures and entirely new domains of biology.

## Supplementary Information

Additional file 1.

## Data Availability

None
