# Supplementary material for: Spatial omics technologies at multimodal and single cell/subcellular level
Source: Genome Biol. 2022 Dec 13;23:256. doi: 10.1186/s13059-022-02824-6 (PMC9746133; doi:10.1186/s13059-022-02824-6)
Supplement: Supplementary file 1 — Additional file 1. [file 13059_2022_2824_MOESM1_ESM.docx]

Review History

**First round of review**

**Reviewer 1**

**Comments to author:**

The authors provided a review on cutting-edge spatial-omics technologies, which is timely and popular currently. As related approaches continue to flood the field of spatial omics, the quality of reviews must also become more demanding. I've seen a lot of well-organized reviews that have a clear theme and sound logic. However, although the authors summarized a lot of spatial-omics profiling technologies and computational tools, it is difficult to follow without a clear direction to help readers understand the developmental process of this field step by step, which are my biggest concerns about this manuscript. Here are my comments on their review.

Major comments:

1. Key technical development and relationship among technologies in spatial omics should be more intuitive in this review. For example, (1) how did the image-based methods increase the number of target genes step by step? What multiplexing strategy used and how to eliminate detection error during multiplexing? (2) how did the slide-based methods narrow the profiling area to improve the resolution? What spatial barcoding strategy used? did the UMI number and detected genes decreased? (3) how did the user-directed profiling methods like LCM-seq, GEO-seq, and DSP, etc. scale up the samples? (4) how did current methods increase the detection modals (ATAC, proteome) instead of focusing on only transcriptome? and (5) Was any strategy in one technology successfully used in another? The authors can compare the specific indexes of each stage of the continuous updated technologies such as (merfish to merfish+), (seqfish to seqfish+), (slide-seq to slide-seq v2), (ST and HDST), (DBiT-seq and Spatial-CUT&Tag), etc.

2. The authors focus on technologies with cellular and subcellular resolution, please state the definition of subcellular resolution. As described in the submitted manuscript, several slide-based technologies are labeled as subcellular resolution. Is there any classification standard for these technologies? For example, based on the spot diameter comparing with average cell size? As we know, 10X Visium (55 μm) and ST (100 μm) are regarded as regional resolution without doubt because each spot contains a mixture of cells. However, even though the spot size is down to nanometer scale, each spot can also contain transcripts from different cells. Can we just treat them as subcellular resolution? Moreover, what is the advantage of these so-called subcellular technologies compared with traditional slide-based approaches except for a higher resolution? Refined cell clustering?

3. The authors also listed computational methods for the analysis of spatially resolved omics. The manuscript should be organized more logically. For example, I want to know how did these in silico methods help to improve existing technologies? For slide-based technologies, achieving single-cell resolution is the top target. Related methods such as CellTrek, MUSE, Cell2location, etc. should be included. For image-based technologies, gene imputation methods should also be described. Again, all listed methods, experimental or computational, should be described in relation to each other.

4. Spatially resolved omics technologies have provided unprecedented insight in biological clinical field. their application should be reviewed comprehensively. This is quite important because all technologies aspire to scientific transform. For example, 10X Visium, DSP, ST, etc. are successfully applied in clinical use. Merfish and seqFish are still difficult in instrumentalization. It is promising to see the extensive use of these technologies, thus, it is needed to organize this review article well.

5. For readers and users, wise choice of an appropriate technology is important for their own research. The authors need to provide guidance on which technologies to use in different situations. For example, frozen samples, FFPE samples, infrequent samples, etc.

However, I didn't see these important questions been addressed in the manuscript.

Minor comments:

The authors should thoroughly check their spelling and consistency of words. For example:

1. The word 'in situ' is used inconsistently throughout the manuscript, with italics in places and without, and 'in-situ' in line 5. Please check thoroughly and rewrite to a uniform format.

2. Similar examples as 'high-throughput sequencing' (high throughput), 'single-cell' (single cell) and 'spatial-omics technologies' (spatial technologies). If the abbreviations have been stated, the rest of the paper should use the abbreviations. PS, most publications use NGS (next-generation sequencing), RNA-seq, or deep sequencing instead of HTS (high-throughput sequencing).

3. In line 38: 'spatial-mics' should be corrected to 'spatial-omics'.

4. In line 136: 'Sample prep and experimental design', although most readers understand the meaning of 'prep', it is required to avoid colloquial language in your writing.

Having so many small problems in the first section of the 'main text' greatly reduces the reader's motivation to stick with it.

**Reviewer 2**

**Comments to author:**

Recently, there has been a lot of exciting developments in the area of Spatial-omics; therefore, the review is timely and appropriate. The review is organized in a way where investigators can obtain an overview of these developments. The reviewer has several suggestions.

Comments:

(1) The title is "spatial-omics technologies at multimodal and subcellular level". There has been thorough and rich description about the usage and applications of single cell resolution or at the tissue level; however, how subcellular resolution spatial omics can be useful for biological analysis is rather very briefly described. Indeed, most analyses described in the paper focuses on single cell or tissue level, not subcellular level. With this regard, the authors should consider revising the title as "single-cell and subcellular level" to be consistent with the content of the review.

(2) The authors did not discuss about spatial single cell methods, such as ZIP-Seq, XYZeq and sci-Space. However, these methods could have many advantages over other spatial transcriptomic methods since they can distinguish single cell omics features more confidently compared to other methods where single cell boundary detection could be highly challenging (especially around the cell surface). The strength and weaknesses of these methods would need to be discussed rather than being ignored.

(3) Regarding multiplexed detection of transcriptome and proteome, SM-omics (https://doi.org/10.1038/s41467-022-28445-y), SPOTS (https://doi.org/10.1101/2022.03.15.484516) and spatial-CITE-seq (https://doi.org/10.1101/2022.04.01.486788) would need to be discussed.

(4) Regarding subcellular resolution, the authors missed two recent high-resolution technologies, Seq-Scope (https://doi.org/10.1016/j.cell.2021.05.010) and Pixel-Seq (https://doi.org/10.1101/2021.03.17.435795). Among these, Seq-Scope showed subcellular analysis of spatial nuclear-cytoplasmic fractionation, which should be relevant to the topic of this review.

(5) Spatial ATAC and Spatial Cut&Tag would need to be discussed in multi-modal analysis, rather than in conclusion. Current discussions only included DBiT-Seq-based technologies, but it should also include Visium-based approach (https://doi.org/10.1101/2022.07.27.500203). In addition, there are two recent preprints showing that spatial omics platforms can also capture microbiome sequences (https://doi.org/10.1101/2022.07.18.500470 and https://doi.org/10.1101/2022.07.18.496977). It would be interesting to discuss these in the context of formerly available FISH-based microbiome detection methods, which provide higher resolution.

**Authors Response**

**Point-by-point responses to the reviewers’ comments:**

Reviewer #1: The authors provided a review on cutting-edge spatial-omics technologies, which is timely and popular currently. As related approaches continue to flood the field of spatial omics, the quality of reviews must also become more demanding. I've seen a lot of well-organized reviews that have a clear theme and sound logic. However, although the authors summarized a lot of spatial-omics profiling technologies and computational tools, it is difficult to follow without a clear direction to help readers understand the developmental process of this field step by step, which are my biggest concerns about this manuscript. Here are my comments on their review.

Major comments:

1. Key technical development and relationship among technologies in spatial omics should be more intuitive in this review. For example, (1) how did the image-based methods increase the number of target genes step by step? What multiplexing strategy used and how to eliminate detection error during multiplexing? (2) how did the slide-based methods narrow the profiling area to improve the resolution? What spatial barcoding strategy used? did the UMI number and detected genes decreased? (3) how did the user-directed profiling methods like LCM-seq, GEO-seq, and DSP, etc. scale up the samples? (4) how did current methods increase the detection modals (ATAC, proteome) instead of focusing on only transcriptome? and (5) Was any strategy in one technology successfully used in another? The authors can compare the specific indexes of each stage of the continuous updated technologies such as (merfish to merfish+), (seqfish to seqfish+), (slide-seq to slide-seq v2), (ST and HDST),

(DBiT-seq and Spatial-CUT&Tag), etc.

Response: We appreciate the reviewer’s suggestions. Overall, we revised our manuscript to cover examples and differences across each technology (i.e., various probe chemistries, barcoding methods, amplification protocols, etc.). We also addressed specific suggestions the reviewer mentioned: (1) We already had spectral and temporal barcoding methods and related literature that discusses in-depth for major technologies. To make it clearer and more complete, we have described how those two barcoding strategies are combined and expanded this in the revised manuscript with examples. We have also added comments on the error correction methods (page 3, lines 93-98), as follows:

“Specifically, high multiplexing is achieved by using spectral barcoding (specific combination of fluorophores each targeting segments of an RNA resolved by microscopy) [22] and temporal barcoding (multiple rounds of probe hybridization and stripping to create predefined color sequence) [23]. Recently, the combination of two barcoding methods and more complicated barcoding strategies can increase the number of molecular entities that can be profiled.”

(2) Most of the spatial profiling methods are slide-based (quantification is done on the tissue slide), and regardless of the methodology type (sequencing or imaging based) nuclei staining is primarily used to segment cells and gain an understanding of the overall tissue morphology. We have updated the manuscript to make these points clear.

(3) We have addressed the reviewer’s question in the updated manuscript by briefly commenting on the multiple ROIs/sample setup for this type of technology. Although we mention LCM-seq, GEO-seq, and DSP in the manuscript as examples of early NGS-based technologies, we did not go deeper given that the scope of the manuscript is on single cell or subcellular resolution technologies. (page 4, lines 143-146):

“LCM-seq [37], geographical position sequencing (Geo-seq) [38], NICHE-seq [39] and NanoString GeoMx DSP [40] are a few additional contemporary examples of this technology. These technologies allow user-directed profiling of specific ROIs with as few as 10 cells, allowing researchers to characterize multiple replicates or tissue types/locations for each sample.”

(4) We have created a new subsection to discuss technologies that enable multi-modal analysis i.e., ATAC, CUT&Tag, metagenomics/DNA, metabolomics, etc. (page 5, lines 183-211)

(5) For the reviewer’s first point, we have included references that incorporate multiple spatial omics methods to address increasing data modalities (i.e., transcriptome and proteome detection) in the updated manuscript (page 4-5, lines 171-181):

“Inspired by these technologies and to overcome the limitations, multiplexed detection methods of transcriptome and proteome have been developed. Examples include Spatial Multi-Omics (SM-Omics) [54], Spatial PrOtein and Transcriptome Sequencing (SPOTS) [55] and spatial co-indexing of transcriptomes and epitopes for multi-omics mapping by NGS (spatial-CITE-seq) [56]. These technologies combine existing NGS-based methodologies to allow computational reconstruction of spatial full transcriptome and 200+ proteome maps. Although these technologies make use of NGS technologies to cover the full transcriptome while recording large panels of proteins in tissues, the full transcriptome characterizations are still limited by resolution (SM-Omics uses 10X Visium which allows 55 µm resolution), or location detection (SPOTS and spatial-CITE-seq uses CITE-seq which is an antibody-binding based method to provide cellular context, not precise locations within the tissue).”

We also created a new section called “Other spatial omics technologies for multi-modal studies” in the updated manuscript to cover technologies that provides epigenome, metabolome, and metagenome characterizations (page 5, lines 183-211). To the second point, regarding updated versions of the technology, for most of the techniques mentioned by the reviewer we only included the latest or the most relevant versions of technology. We do cite references to the original technologies in the legend of **Table 1**. We also added descriptions of developments in spatial barcoding strategies in the updated manuscript (page 3, lines 96-102):

“Recently, the combination of two barcoding methods and more complicated barcoding strategies can increase the number of molecular entities that can be profiled. For example, while seqFISH uses spectral barcoding of genes across four or five fluorophores for each given temporal barcode, seqFISH+ adds mRNA-specific sequences to assign a pseudocolor for each spectral barcode (fluorophore). The new seqFISH+ technology can now capture 60 pseudocolors instead of 5, allowing 8000 gene profiles each cycle [24].”

2. The authors focus on technologies with cellular and subcellular resolution, please state the definition of subcellular resolution. As described in the submitted manuscript, several slide-based technologies are labeled as subcellular resolution. Is there any classification standard for these technologies? For example, based on the spot diameter comparing with average cell size? As we know, 10X Visium (55 μm) and ST (100 μm) are regarded as regional resolution without doubt because each spot contains a mixture of cells. However, even though the spot size is down to nanometer scale, each spot can also contain transcripts from different cells. Can we just treat them as subcellular resolution? Moreover, what is the advantage of these so-called subcellular technologies compared with traditional slide-based approaches except for a higher resolution? Refined cell clustering?

Response: We have updated our manuscript to include definition and details of subcellular resolution as less than 10 µm (page 2, line 46; lines 49-54), as follows:

“However, at <10 µm resolution, the cell body and nucleus can be detected for single cell level quantification; with technologies that allow <1 µm resolution, researchers can now detect a few other large organelles including cytoplasm-membrane distinction; at 200-300 nm, more well-resolved characterizations are possible including mitochondria-, ER-, Golgi- specific transcript or protein quantifications. At 50 nm ranges, entirely new cellular phenotypes (e.g., movement of organelles and protein trafficking) can be measured.”

We agree with the reviewer that each spot can contain transcripts from different cells especially for technologies like Slide-seqV2 or DBiT-seq (at resolution of 10 µm) and therefore near-single cell resolution rather than a true subcellular resolution. Technologies such as FISSEQ or Stereo-seq (resolution of 600 and 500 nm, respectively) are fine enough to capture 250 to 400 spots per single cell on average (assuming 10 µm diameter cell) if one were to aggregate the post-filtered counts (after filtering undetermined spots) into a form of a single cell count matrix.

3. The authors also listed computational methods for the analysis of spatially resolved omics. The manuscript should be organized more logically. For example, I want to know how did these in silico methods help to improve existing technologies? For slide-based technologies, achieving single-cell resolution is the top target. Related methods such as CellTrek, MUSE, Cell2location, etc. should be included. For image-based technologies, gene imputation methods should also be described. Again, all listed methods, experimental or computational, should be described in relation to each other.

Response: We have added these methods into the updated manuscript (page 10, lines 436-441), as follows:

“Recently, more packages and methodologies, such as Cell2location [116], CellTrek [117], multi-modal structured embedding (MUSE) [118], and Tangram [119], are being developed specifically to map single cell information to spatial omics analyses. For example, Tangram aligns expression profiles from sc/snRNA-seq to spatial datasets from the same region including MERFISH, STARmap, general smFISH, Visium and histological images [119].”

To the reviewer’s point about methodologies for slide-based technologies and image-based technologies, we believe they are similar to each other given that the technologies highlighted in the review are strictly at the single cell or subcellular resolution level. For slide-based technologies such as 10X Visium, ST, or DSP, it is crucial to use technologies that achieve single cell resolution; however, the technologies that allow detection in single cell or lower resolution do not have this problem and therefore are treated similarly to image-based methods. Lastly, we have updated our manuscript to explicitly define and discuss gene imputation methods, from already discussed methods that allows sc/snRNA-seq and other data integration.

4. Spatially resolved omics technologies have provided unprecedented insight in biological clinical field. their application should be reviewed comprehensively. This is quite important because all technologies aspire to scientific transform. For example, 10X Visium, DSP, ST, etc. are successfully applied in clinical use. Merfish and seqFish are still difficult in instrumentalization. It is promising to see the extensive use of these technologies, thus, it is needed to organize this review article well.

Response: We appreciate the reviewer’s feedback here and agree that some technologies have not been highly implemented in clinical settings. Much of this is due to (1) limited throughput of the technologies to scale the level needed for routine clinical use (including ease of library design, instrumentalization, and cost), (2) rigorous testing in settings with clearly defined clinical endpoints, and (3) lack of standard analysis options after data collection - particularly due to novelty of the data. We feel that this is slightly out of the scope of the review since many of the technologies also depend on the business model each company builds; some of the technologies are still in the phase of product development and not available. However, we have updated our manuscript to cover the importance of analysis packages for clinical use applications (page 7, lines 289-294; pages 10-11, lines 456-458).

5. For readers and users, wise choice of an appropriate technology is important for their own research. The authors need to provide guidance on which technologies to use in different situations. For example, frozen samples, FFPE samples, infrequent samples, etc.

However, I didn't see these important questions been addressed in the manuscript.

Response: We have added these points in the revised manuscript. Briefly, flash frozen samples are most preferred and compatible with most technologies, however FFPE samples are sometimes used for practical reasons (availability, storage and ease of processing). We have copied the updated portion of the manuscript below (pages 5-6, lines 219-224):

“Most of the techniques offer compatibility with flash frozen (FF) and formalin-fixed paraffin embedded (FFPE) formats. FF format often yields better RNA quality and simpler extraction processing; however, FFPE format more faithfully conserves tissue architecture and is easier to store and ship. Success also varies depending on the sample quality (RNA integrity and processing protocols) and technology (probe/antibody design, permeabilization and chemistry for hybridization, imaging, and library preparation).”

Minor comments:

The authors should thoroughly check their spelling and consistency of words. For example:

1. The word 'in situ' is used inconsistently throughout the manuscript, with italics in places and without, and 'in-situ' in line 5. Please check thoroughly and rewrite to a uniform format.

Response: We have checked the manuscript and formatted uniformly with italics.

2. Similar examples as 'high-throughput sequencing' (high throughput), 'single-cell' (single cell) and 'spatial-omics technologies' (spatial technologies). If the abbreviations have been stated, the rest of the paper should use the abbreviations. PS, most publications use NGS (next-generation sequencing), RNA-seq, or deep sequencing instead of HTS (high-throughput sequencing).

Response: We appreciate the reviewer’s detailed comments. We have checked the manuscript to ensure we have consistent terminology and use of abbreviations.

3. In line 38: 'spatial-mics' should be corrected to 'spatial-omics'.

Response: We apologize for this mistake. This typo has been corrected.

4. In line 136: 'Sample prep and experimental design', although most readers understand the meaning of 'prep', it is required to avoid colloquial language in your writing.

Response: We thank you for this suggestion - we have modified the subsection name.

Having so many small problems in the first section of the 'main text' greatly reduces the reader's motivation to stick with it.

Reviewer #2: Recently, there has been a lot of exciting developments in the area of Spatial-omics; therefore, the review is timely and appropriate. The review is organized in a way where investigators can obtain an overview of these developments. The reviewer has several suggestions.

(1) The title is "spatial-omics technologies at multimodal and subcellular level". There has been thorough and rich description about the usage and applications of single cell resolution or at the tissue level; however, how subcellular resolution spatial omics can be useful for biological analysis is rather very briefly described. Indeed, most analyses described in the paper focuses on single cell or tissue level, not subcellular level. With this regard, the authors should consider revising the title as "single-cell and subcellular level" to be consistent with the content of the review.

Response: We appreciate the reviewer’s comment and suggestions. We agree with the reviewer that most of the analysis are focused on the single cell level, and it is because there is not much done on the usage of the subcellular resolution other than few FISH-based studies visualizing viral proteins and their movements. We have revised the title to include single cell so that the content of the review is properly reflected.

(2) The authors did not discuss about spatial single cell methods, such as ZIP-Seq, XYZeq and sci-Space. However, these methods could have many advantages over other spatial transcriptomic methods since they can distinguish single cell omics features more confidently compared to other methods where single cell boundary detection could be highly challenging (especially around the cell surface). The strength and weaknesses of these methods would need to be discussed rather than being ignored.

Response: We have previously commented on CITE-seq, ZipSeq, ExSeq, XYZeq that spatial barcoding methods lose the organization context and therefore are not included in the manuscript. However, we do agree that those technologies allow the users to distinguish single cell features more confidently. We have not included Sci-Space though, because the resolution of the spatial coordinates is 200 μm. We have updated the manuscript to incorporate this (page 4, lines 167-171):

“In addition, there are spatial barcoding methods such as CITE-seq [50], ZipSeq [51], ExSeq [45], XYZeq [52], or sci-space [53], where additional cellular information such as antibody staining or barcoding is added before pooling for single cell sequencing workflow. Additional single cell features such as cell types or tissue location or compartmentalization can be deduced; however, a precise picture of cellular organization is not yet possible.”

(3) Regarding multiplexed detection of transcriptome and proteome, SM-omics (https://www.nature.com/articles/s41467-022-28445-y), SPOTS (https://www.biorxiv.org/content/10.1101/2022.03.15.484516v1) and spatial-CITE-seq (https://www.biorxiv.org/content/10.1101/2022.04.01.486788v1) would need to be discussed.

Response: We agree with the reviewer and have updated our manuscript to include these references (pages 4-5, lines 171-181). In the revised manuscript:

“Inspired by these technologies and to overcome the limitations, multiplexed detection methods of transcriptome and proteome have been developed. Examples include Spatial Multi-Omics (SM-Omics) [54], Spatial PrOtein and Transcriptome Sequencing (SPOTS) [55] and spatial co-indexing of transcriptomes and epitopes for multi-omics mapping by NGS (spatial-CITE-seq) [56]. These technologies combine existing NGS-based methodologies to allow computational reconstruction of spatial full transcriptome and 200+ proteome maps. Although these technologies make use of NGS technologies to cover the full transcriptome while recording large panels of proteins in tissues, the full transcriptome characterizations are still limited by resolution (SM-Omics uses 10X Visium which allows 55 µm resolution), or location detection (SPOTS and spatial-CITE-seq uses CITE-seq which is an antibody-binding based method to provide cellular context, not precise locations within the tissue).”

(4) Regarding subcellular resolution, the authors missed two recent high-resolution technologies, Seq-Scope (https://www.cell.com/cell/fulltext/S0092-8674(21)00627-9?_returnURL=https%3A%2F%2Flinkinghub.elsevier.com%2Fretrieve%2Fpii%2FS0092867421006279%3Fshowall%3Dtrue) and Pixel-Seq (https://www.biorxiv.org/content/10.1101/2021.03.17.435795v1). Among these, Seq-Scope showed subcellular analysis of spatial nuclear-cytoplasmic fractionation, which should be relevant to the topic of this review.

Response: We appreciate the reviewer’s comment and have included these technologies in the revised manuscript (page 4, lines 148-154):

“Technologies such as Fluorescent In Situ Sequencing (FISSEQ) [41], spatially-resolved transcript amplicon readout mapping (STARmap) [42], Slide-SeqV2 [43], deterministic barcoding in tissue for spatial omics sequencing (DBiT-seq) [44], expansion sequencing (ExSeq) [45], High-Definition Spatial Transcriptomics (HDST) [46], Seq-scope [47], polony (or DNA cluster)-indexed library-sequencing (PIXEL-seq) [48], and SpaTial Enhanced REsolution Omics-Sequencing (Stereo-Seq) [49] (and ST, 10x Visium for multi-cell version of the same technology) use grid-like nanoballs or sequencing sites on the slide.”

(5) Spatial ATAC and Spatial Cut&Tag would need to be discussed in multi-modal analysis, rather than in conclusion. Current discussions only included DBiT-Seq-based technologies, but it should also include Visium-based approach (https://www.biorxiv.org/content/10.1101/2022.07.27.500203v1.full). In addition, there are two recent preprints showing that spatial omics platforms can also capture microbiome sequences (<https://www.biorxiv.org/content/10.1101/2022.07.18.500470v1> and https://www.biorxiv.org/content/10.1101/2022.07.18.496977v1). It would be interesting to discuss these in the context of formerly available FISH-based microbiome detection methods, which provide higher resolution.

Response: We agree with the reviewer’s comment and have created new subsection as “Other spatial technologies for multi-modal study.” We have included spatial epigenomics as well as spatial microbiome characterization as reviewer suggested. We also added spatial metabolomics and metabolomic (DNA level) characterizations which are interesting and relevant in our discussion of multimodal studies possible with spatial technologies.

The updated manuscript is copied below (page 5, lines 183-211):

“*Other spatial omics technologies for multi-modal study*

Similar to the spatial omics technologies introduced above, which mainly focus on gene expression profiles (and surface marker proteins), approaches around spatial genomics, metabolomics, metagenomics, and epigenomics are also emerging. Spatial ATAC sequencing can be performed by in situ Tn5 transposition, and probe ligation using microfluidics devices, followed by standard digestion and sequencing for chromatin accessibility profiling [57, 58]. Resolution is limited by the microfluidic channel width (20 µm); however, single cell resolution is less crucial than transcript quantification as the analysis relies on the signals within the nuclear regions. Similarly, specific chromatin modifications can be quantified using Spatial-CUT&Tag that applies CUT&Tag chemistry with microfluidic devices [59]. Both technologies use deterministic barcoding delivered over the tissue surface through a microfluidic device attached to the slide. The barcodes are delivered twice perpendicularly so that the combinations result in 2D arrayed pixels containing spatial information. Spatial metabolomics techniques such as targeted approaches using antibodies (metaFISH) or untargeted using matrix-assisted laser desorption/ionization imaging mass spectrometry (MALDI-IMS) hold promise for mapping the spatial context of metabolic species and molecular interactions within the native tissue context, but still suffer from trade-offs in spatial resolution or the breadth of molecular entities profiled [60].

Such added layers of genomic data allow researchers to ask new biological questions. For example, in addition to expression level changes within tissue microenvironment, clonal expansion of specific mutations and spatial co-occurrences can be investigated using spatial genomics such as slide-DNA-seq [61]. This method is a modified version of slide-seq where DNA sequences are captured with small (3 mm) beads that are spatially indexed, instead of RNA transcripts. Optimized methodologies for histone removal and Tn5 treatments for a variety of tumor tissue types have been shown to prevent potential bias in DNA capture. More recently, spatial host-microbiome sequencing (SHM-seq) has been reported [62]. SHM-seq is an adapted version from Spatial Transcriptomics, where mRNA probes for transcript captures are modified to DNA capture probes so that they can obtain both polyadenylated transcripts and 16S rRNA hypervariable regions. The recent progress on spatial characterizations allows researchers to locate interactions at the genomics, cellular, and organismal level [63].”

**Second round of review**

**Reviewer 1**

In the revised manuscript, Park et al. modified their manuscript extensively, which fully addressed all my concerns. I have no further questions. However, before moving on to the next step, I suggest the authors thoroughly check their language and spelling. Second, the manuscript needs to be formatted well. For example, flaws still remain in Table 2. Third, all figures must be original. Please avoid any downloaded or third-party material. The authors must state this.

**Reviewer 2**

The responses are satisfactory and the manuscript has been improved.
